# Supplementary material for: Translational Frameshifting in the chlD Gene Gives a Clue to the Coevolution of the Chlorophyll and Cobalamin Biosyntheses
Source: Microorganisms. 2022 Jun 11;10(6):1200. doi: 10.3390/microorganisms10061200 (PMC9227772; doi:10.3390/microorganisms10061200)
Supplement: Supplementary file 1 [file microorganisms-10-01200-s001.zip › microorganisms-1698104-supplementary.pdf]

Supplementary Materials

Translational frameshifting in the *chlD* gene gives a clue to the co-evolution of the chlorophyll and cobalamin biosyntheses

Stepan Kuznetsov[1], Alexander Milenkin[1] and Ivan Antonov [2,3,*]

[1] Moscow Institute of Physics and Technology, Dolgoprudny, Moscow Region, Russia
[2] Institute of Bioengineering, Research Center of Biotechnology, Russian Academy of Science, Moscow, Russia;
[3] Laboratory of Bioinformatics, Faculty of Computer Science, National Research University Higher School of Economics, Moscow, Russia

Supplementary Figures

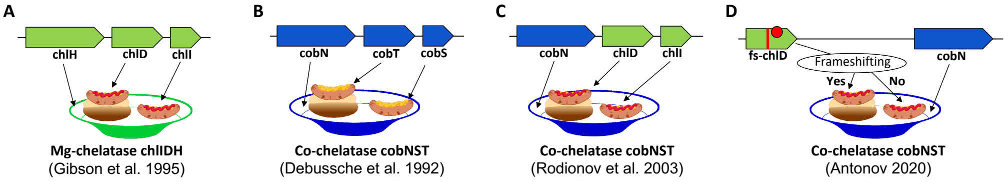

Supplementary Figure S1. The main strategies utilized by different prokaryotic species to encode Mg-and Co-chelatases. For visualization purposes, a chelatase is depicted as a sausage (small subunit) and a hot dog (medium subunit) on a plate (large subunit). This representation highlights that facts that each of the chelatases consists of three different subunits and that the small subunit is similar to the N-terminal part of the medium subunit (in other words "a sausage is a part of a hot dog").

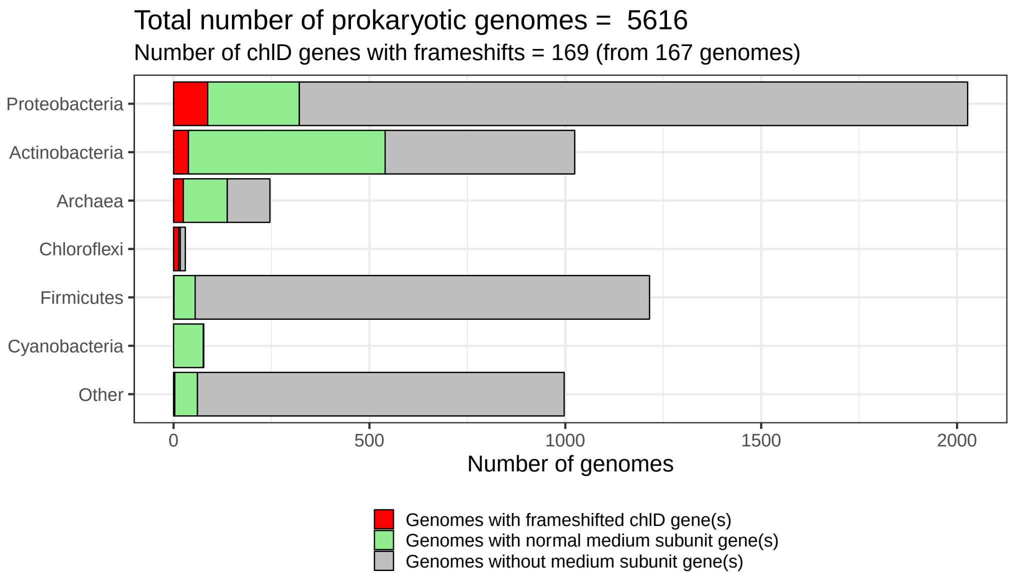

Supplementary Figure S2. The number of the identified chlD genes in the genomes from different prokaryotic phyla. In each group the fraction of the chlD genes with predicted -1 and +1 frameshift is indicated by the red and green colors, respectively.

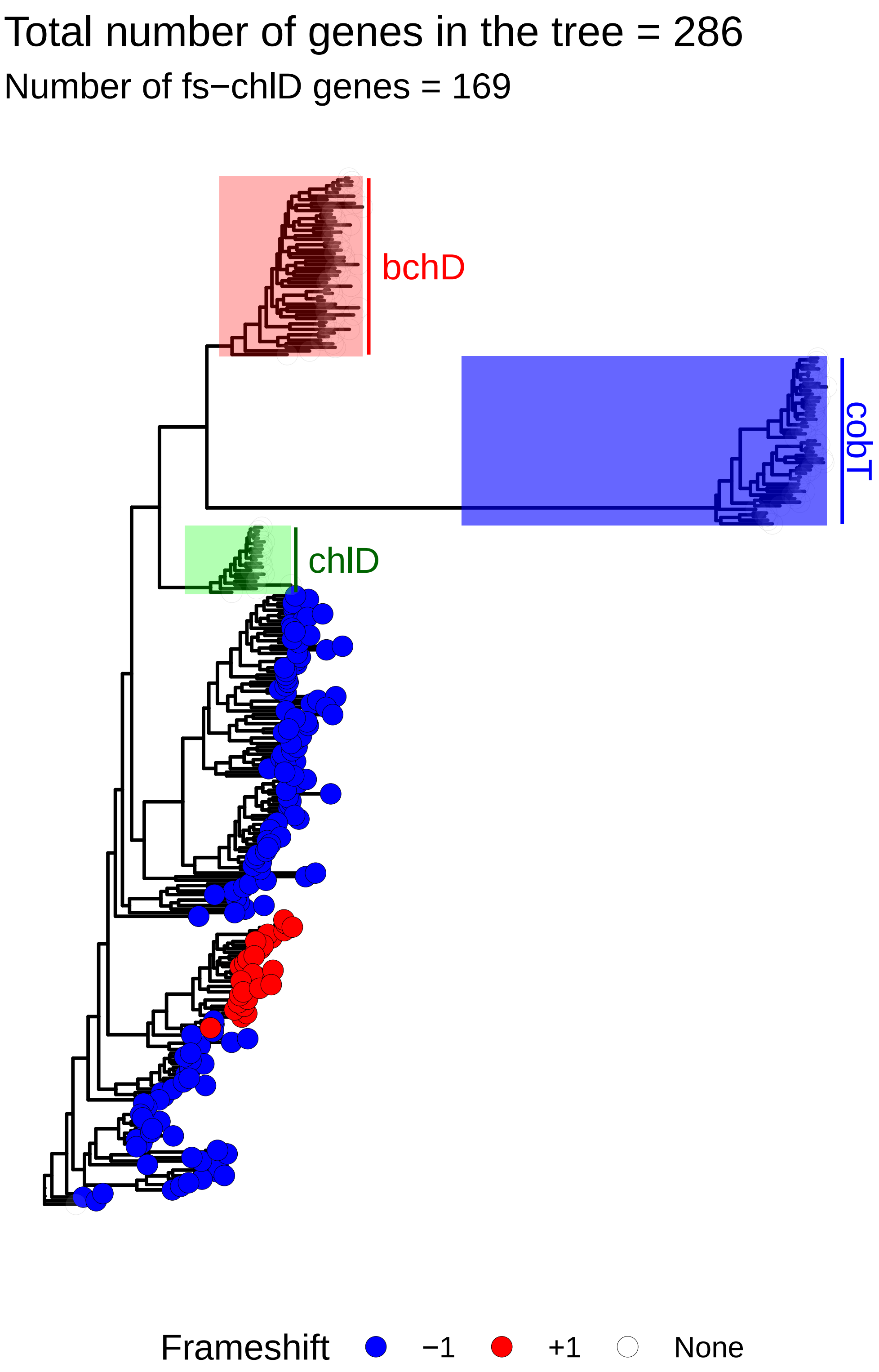

Supplementary Figure S3. The reference phylogenetic tree of the annotated medium chelatase subunit proteins and as well as the long products of the fs-chlD genes. The only annotated archaeal bchD gene (METFOR_RS04410 from Methanoregula formicica SMSP) was used as the outgroup. The -1 and +1 frameshift types in the fs-chlD genes are indicated by the blue and red colors, respectively.

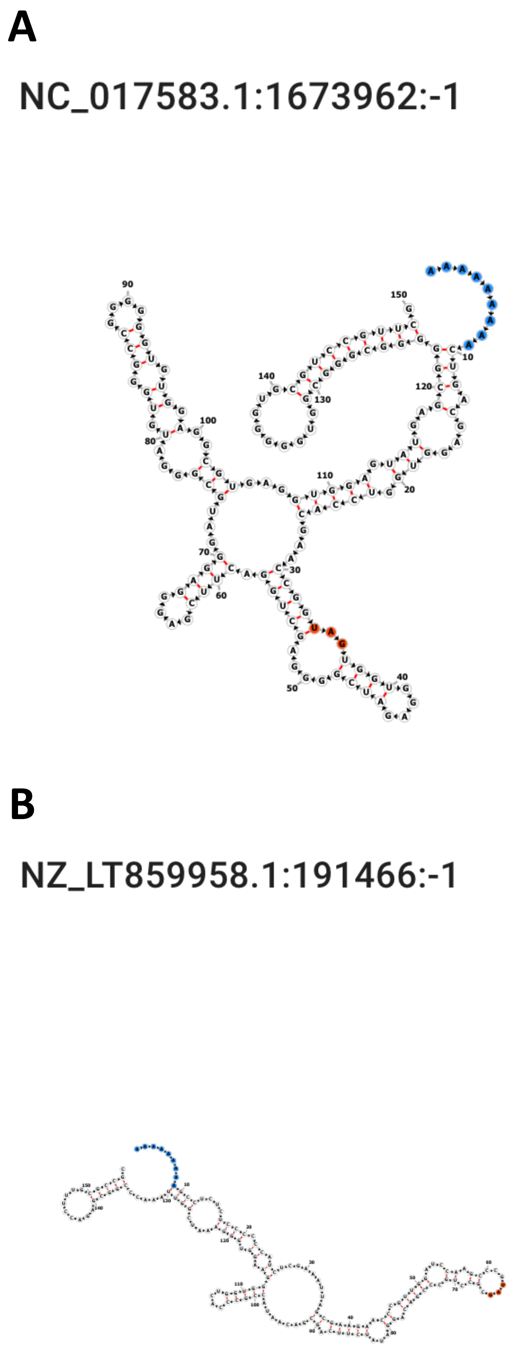

Supplementary Figure S4. Frameshifting signlas identified in the fs-chlD genes from Spirochaeta thermophila DSM 6578 (A) and Brevefilum fermentans (B). The slippery site of the signal and the in-frame stop codon are marked by the blue and red colors, respectively. The coordinates of the predicted -1 frameshifts are NC_017583.1:1673962 and NZ_LT859958.1:191466.

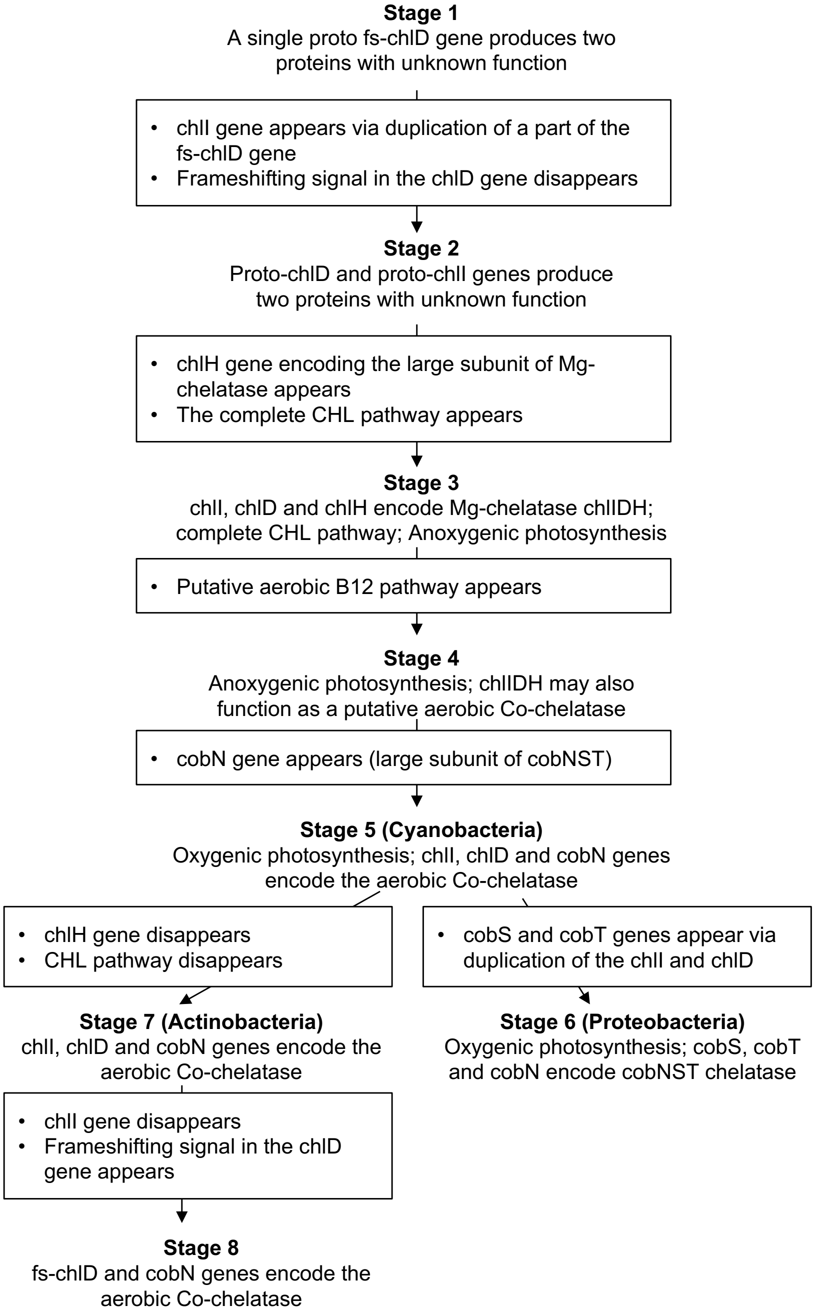

Supplementary Figure S5. A putative chain of events that could happen between different stages during the evolution of the CHL and aerobic B12 pathways.
